# Conflict between Dolphins and a Data-Scarce Fishery of the European Union

**DOI:** 10.1007/s10745-018-9989-7

**Published:** 2018-03-27

**Authors:** Robin Thomas Ernest Snape, Annette Cameron Broderick, Burak Ali Çiçek, Wayne John Fuller, Nicholas Tregenza, Matthew John Witt, Brendan John Godley

**Affiliations:** 1 Centre for Ecology and Conservation, College of Life and Environmental Sciences, University of Exeter, Penryn Campus, Cornwall, TR10 9FE UK; 2 Society for Protection of Turtles, PK.65, Mersin 10 Kyrenia, North Cyprus Turkey; 3 Underwater Research and Imaging Centre, Biological Sciences Department, Eastern Mediterranean University, Mersin 10 Famagusta, North Cyprus Turkey; 4 Faculty of Veterinary Medicine, Near East University, 10 Mersin, Turkey; 5 Chelonia Limited, The Barkhouse, North Cliff, Mousehole, Cornwall, TR19 6PH UK; 6 Environment and Sustainability Institute, University of Exeter, Penryn Campus, Cornwall, TR10 9FE UK

**Keywords:** Dolphins, Depredation, Net damages, Economic losses, Fisheries overexploitation, Fisheries management, Northern Cyprus, Mediterranean

## Abstract

Fisheries depredation by marine mammals is an economic concern worldwide. We combined questionnaires, acoustic monitoring, and participatory experiments to investigate the occurrence of bottlenose dolphins in the fisheries of Northern Cyprus, and the extent of their conflict with set-nets, an economically important metier of Mediterranean fisheries. Dolphins were present in fishing grounds throughout the year and were detected at 28% of sets. Net damage was on average six times greater where dolphins were present, was correlated with dolphin presence, and the associated costs were considerable. An acoustic deterrent pinger was tested, but had no significant effect although more powerful pingers could have greater impact. However, our findings indicate that effective management of fish stocks is urgently required to address the overexploitation that is likely driving depredation behaviour in dolphins, that in turn leads to net damage and the associated costs to the fisheries.

**Electronic supplementary material:**

The online version of this article (10.1007/s10745-018-9989-7) contains supplementary material, which is available to authorized users.

## Introduction

Reports of increasing catch depredation among odontocetes and pinnipeds reflect the expansion, intensification, and diversification of world fisheries and a wide range of marine mammal species depredate in a diversity of fishing gears (Read *et al*. 2006). For example, seals with gillnet sets in northern Europe (Cosgrove *et al*. 2015), killer whales with demersal longlines in the South Atlantic (Guinet *et al*. 2015), and false killer whales with pelagic longlines in the Pacific (Forney *et al*. 2011). Fisheries may face considerable economic losses through spoil of catch and destruction of gear (Brotons *et al*. 2008a; Read 2008) whilst even low levels of marine mammal mortality through bycatch (accidental or unintended catch: Brotons *et al*. 2008b; Lauriano *et al*. 2009; Read 2008) or injury associated with depredation (Gomerčić *et al*. 2009) can cause concerning marine mammal population declines (D’Agrosa *et al*. 2000; Lewison *et al*. 2004).

It is important to understand the nature of marine mammal depredation interactions in order to both provide adequate protection of threatened marine mammal species and to support fishing economies. The latter is particularly relevant in small-scale fisheries (SSF) because they typically support large numbers of fishers compared with more industrialised fisheries (Alfaro-Shigueto *et al*. 2010; Jacquet and Pauly 2008) and the economic impact of depredation is shouldered more directly by individuals rather than corporations.

The Mediterranean Sea is a global marine biodiversity hotspot (Bianchi and Morri 2000; Coll *et al*. 2010, 2011). It is also heavily impacted by fisheries, (Costello *et al*. 2010; Micheli *et al*. 2013; Selig *et al*. 2014) with 52% percent of stocks overfished, compared to 29% worldwide (Food and Agricultural Organisation of the United Nations [FAO] 2014). Furthermore, studies across six countries that collectively constitute over half of the region’s landings (FAO 2016), indicate that catches are greatly underestimated (Coll *et al*. 2014; Pauly *et al*. 2014; Piroddi *et al*. 2015; Ulman *et al*. 2014, 2015). Polyvalent, small-scale vessels make up 80% of the Mediterranean fleet and chiefly use set nets such as gillnets and trammel nets (General Fisheries Commission for the Mediterranean [GFCM] 2016). Among these fisheries little information is available regarding production volumes or bycatch rates (GFCM 2016). With its rich biodiversity and large numbers of SSF fleets, the Mediterranean thus provides a useful laboratory for examining interactions between SSF and threatened marine fauna.

There is a relative paucity of information regarding the nature and extent of interactions between bottlenose dolphins (*Tursiops truncates*) (hereafter referred to as dolphin) and Mediterranean SSF (GFCM 2016), yet depredation in set nets is reported across the region (Spain: Brotons *et al*. 2008a, 2008b; Gazo *et al*. 2008; France: Rocklin *et al*. 2009; Italy: Bearzi *et al*. 2011, Blasi *et al*. 2015, Buscaino *et al*. 2009; Díaz López 2006; Lauriano *et al*. 2009; Maccarrone *et al*. 2014; Pennino *et al*. 2015; Croatia: Gomerčić *et al*. 2009; Greece: Gonzalvo *et al*. 2015; Turkish Black Sea coast: Gönener and Özdemir 2012; Turkish Mediterranean coast: Ali Cemal Gücü, personal communication, February 4, 2016; Cyprus: Dawson *et al*. 2013; Libya: Ibrahim Benamer, personal communication, February 4, 2016; Tunisia: Aydi *et al*. 2013). Among these reports, some efforts have been made to assess rates of dolphin depredation and economic damage, but due to variation in parameters measured and in methods employed among studies^1^ it is often difficult to draw comparisons between fisheries and dolphin populations. In all cases, dolphins are a subject of complaint by fishers as they spoil catch and damage nets. In Spain, Italy, and France studies in commercial fisheries have estimated the annual cost in terms of damage to catch to be €1000–€2000 per vessel or 6.5–8.3% of catch value (Brotons *et al*. 2008b; Gazo *et al*. 2008; Lauriano *et al*. 2004; Rocklin *et al*. 2009). Italian (Bearzi *et al*. 2011) and Greek (Gonzalvo *et al*. 2015) fishers claim that dolphin depredation costs from €500 to €20,000 per vessel annually.

In the Balearic Islands and Sardinia dolphin bycatch in set nets as a result of depredation (Brotons *et al*. 2008a) is considered to have serious conservation implications (Bearzi *et al*. 2012; Brotons *et al*. 2008b; Díaz López 2006). In Croatia, 10% of stranded dolphins showed laryngeal strangulation resulting from swallowing sections of set net (Gomerčić *et al*. 2009), indicating an additional source of mortality. Given the vulnerable conservation status of the Mediterranean bottlenose dolphin subpopulation (Bearzi *et al*. 2012), the apparent basin-wide geographic extent of the issue and the number of fisher livelihoods affected (of the order of 150,000 polyvalent fishers; GFCM 2016) assessing and mitigating dolphin interactions with set net fisheries should be a priority from both a conservation and economic perspective.

A number of studies into mitigating dolphin depredation in Mediterranean set net fisheries have focussed on acoustic deterrent devices called ‘pingers’ as a means of reducing depredation interactions. Pingers emit sounds at frequencies and intensities that are intended to discourage the approach of cetaceans (reviewed by Dawson *et al*. 2013). In these trials, pingers have shown some positive results with 87% reduction in net damage, 49% reduction in interaction rates, and 9% increase in yield in the Balearic Islands (Brotons *et al*. 2008a; Gazo *et al*. 2008). In Italy net damage was reduced by almost a third with 28% higher target catch (Buscaino *et al*. 2009; Maccarrone *et al*. 2014). However, acoustic deterrents do not always provide the intended results (Pirotta *et al*. 2016) and in Tunisia, pingers led to increased dolphin depredation (Aydi *et al*. 2013), suggesting that in some cases they may produce a “dinner bell” effect.

Northern Cyprus has a polyvalent fishery which chiefly uses set nets to land fish that is sold in the north of the island and in the Republic of Cyprus-controlled south through the green line regulation (EU regulation no: 886/2004: http://eur-lex.europa.eu/LexUriServ/LexUriServ.do?uri=CONSLEG:2004R0866:20080627:EN:PDF). However, despite being a *de jure* area of the European Union, since the north operates as a de facto state, the fishery is currently exempt from European Union legislation pending resolution of the Cyprus dispute. Results of questionnaire surveys to build knowledge of conflicts with threatened marine vertebrates in northern Cyprus (Snape *et al*. 2013) indicated net damage resulting from dolphin depredation was the chief economic concern among fishers. However, since no landings data are available for this region of Cyprus (Ulman *et al*. 2014), it is difficult to contextualise and understand the extent of these interactions based on the opinion of fishers alone. In order to gain a better understanding these interactions and to develop mitigation strategies to protect fisher livelihoods we combined a questionnaire survey, acoustic monitoring, and onboard observations. Our aims were to determine a) the seasonal presence of dolphins in fishing grounds, b) the rate of dolphin interactions with set nets, the chief gear type used by this polyvalent fleet (Ulman *et al*. 2014), c) loss of earnings resulting from dolphin interactions in terms of net damage, contextualised against estimated landings, d) the effect of a pinger on acoustic detections, net damage, and landings, and e) the rate of dolphin bycatch and mortality.

## Methods

### Questionnaire Survey

**Fig. 1:**
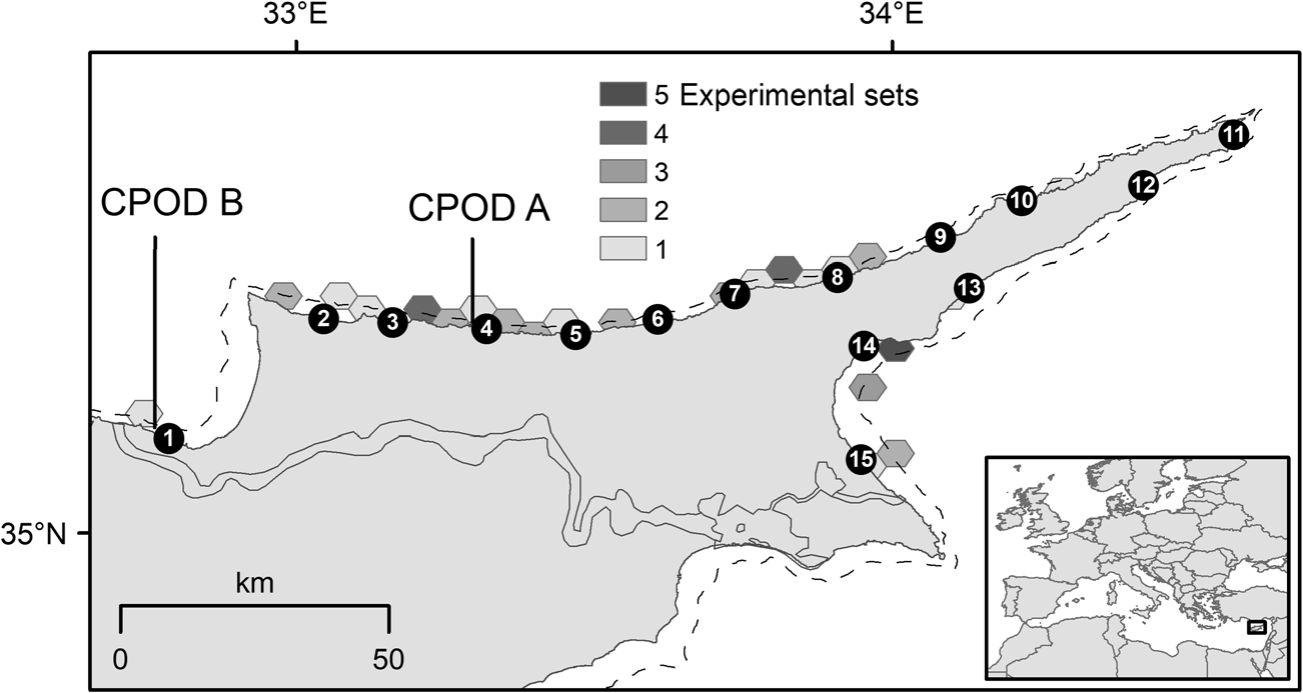
Map of study area. Distribution of experimental sets represented by shaded hexagons (size = 5 km between parallel edges) where tone indicates the number of experiments (*n* = 46) undertaken within in each (see insert key). Black numbered circles indicate fishing harbours. Fishing vessels participating in the study were based at harbours 1, 3, 4, 6, 7, 8, 10, 13, 14 and 15. Arrows indicate the position of static CPODs A and B. Grey broken line indicates the 100 m bathymetric contour. Harbour locations are 1: Gemikonağı, 2: Kayalar, 3: Lapta, 4: Girne, 5: Alagadi, 6: Esentepe, 7: Tatlısu, 8: Kaplıca, 9: Balalan, 10: Yeni Erenköy, 11: Zafer Burnu, 12: Şelonez, 13: Kumyalı, 14: İskele and 15: Mağusa

During 2010 and 2011, 140 captains of fishing vessels were surveyed across the ports of northern Cyprus (Fig. 1). The surveys were designed to provide quantitative information on the most important fishing techniques (see Snape *et al*. 2013), temporal interactions between dolphins and set nets, fishing effort, temporal fishing patterns, and the frequency of dolphin encounters, depredation, bycatch, and mortality events. Fishers were asked the following:

- whether they used set nets and to indicate the months during which their nets were most impacted by dolphins;
- to indicate on average how many fishing trips they made per month from the options 1–10, 11–20, 21–30, 31–40 and > 40;
- during which months they were active, whether they had encountered dolphins during the past year, and whether the dolphins had interfered with their fishing during these encounters;
- the number of dolphins that they generally encounter, whether they had caught any dolphins, if so in which gears and whether those caught had survived.

### Monitoring in Commercial Fisheries

To understand the temporal occurrence of dolphins in coastal fishing grounds and as a reference for comparing rates of dolphin occurrence against those at set nets, two CPODs (passive acoustic monitoring instruments that detect toothed cetaceans by identifying the trains of echolocation sounds they produce; chelonia.co.uk) were moored at Girne (A) and Gemikonağı (B) (Fig. 1) for the periods 1 January to 8 August 2014 and 1 January to 31 December 2014, respectively. CPODs A and B were set at depths of 50 m and 40 m, respectively, which were close to the mean fishing depth recorded during onboard experiments and within fishing areas (Table 1). The CPODs were attached to mooring lines at 2 m above the sea bed and were deployed and serviced using commercial fishing vessels of participating fishers. They were replaced at two-to-four-month intervals with fully charged and serviced CPODs, thus enabling collection of a continuous dataset. All acoustic data were handled in CPOD.exe (www.chelonia.co.uk) where a classification (KERNO classifier) was used to distinguish dolphin click trains from other underwater sounds. Only those click trains that KERNO identified as high and moderate quality were used for analysis (for validity see Tregenza *et al*. 2016).

**Table 1:** Descriptive statistics for soak times and set depths of control and pinger-equipped nets. DPM = Dolphin detection positive minutes, SE = Standard error

		Depth (m)	Soak duration (mins)	CPOD minutes	DPM	% DPM	Net damage (m^2^)	Total haul (kg)
Control	Total		10360	9997	169		27.11	44.4
	Mean	43	225	227	3.8	2.2	0.60	0.97
	SE	3	17	17	2.2	1.4	0.25	0.24
	Range	17-155	36-588	35-589	0-91	0.0-61.1	0.00-10.17	0-10
Pinger-equipped	Total		9275	8987	188		101.64	53.7
	Mean	42	202	200	4.2	1.3	2.26	1.17
	SE	4	16	17	3.3	0.9	1.42	0.27
	Range	16-150	57-592	53-592	0-152	0.0-42.7	0.00-63.12	0-10

**Fig. 2:**
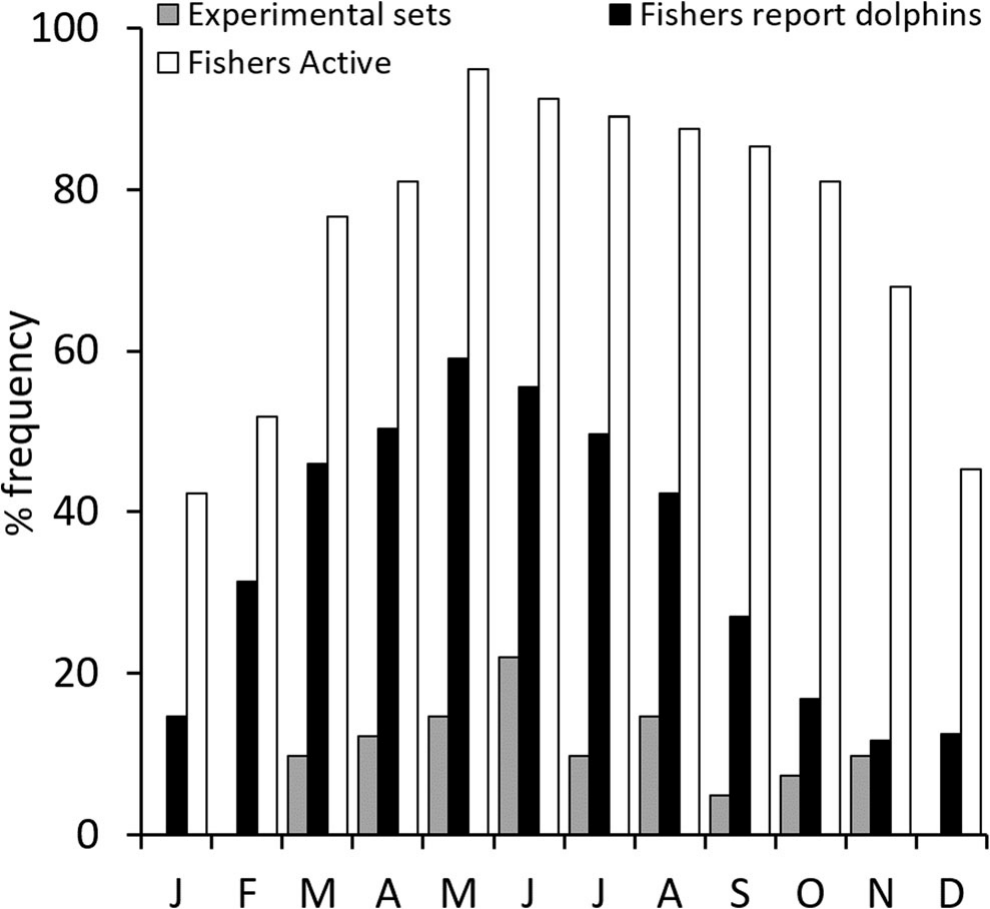
Temporal distribution of experiments (n = 46) made during the study period, opinion of fishers regarding months during which dolphin interactions are most frequently encountered (*n* = 101 respondents) and months that fishers claim to be active (*n* = 124 respondents)

**Fig. 3:**
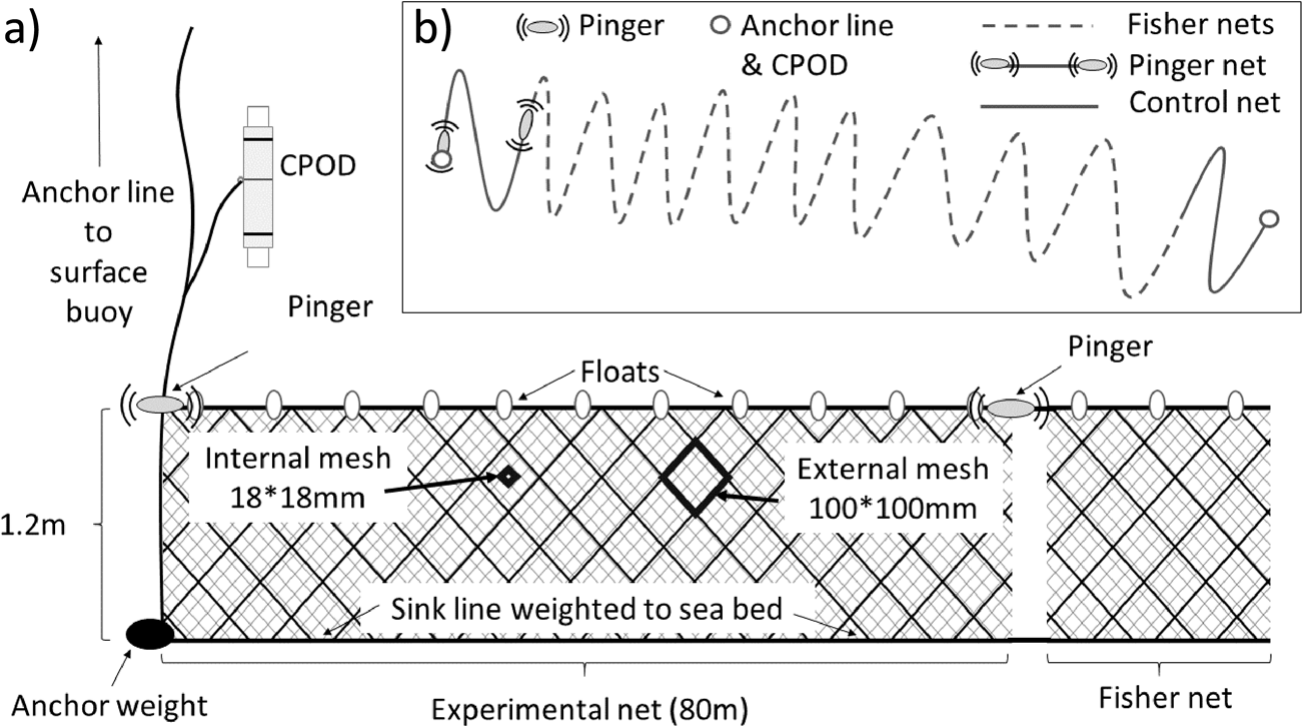
Schematic representation of experimental set net configuration. **a** The vertical configuration of pinger sets. Control set configurations were identical except without pingers. Experimental and control sets were deployed at terminal ends of a larger set of commercial nets deployed by the fisher (see Methods). **b** Typical horizontal placement of nets in relation to each other and to fisher net set. Objects are not drawn to scale

To estimate the frequency and extent of dolphin depredation in set net fisheries, 46 experimental set nets were carried out onboard 14 commercial fishing vessels (<12 m in length), between 15 November 2010 and 17 April 2013. Experimental sets were undertaken from ports across the study region (Fig. 1) and were temporally distributed to reflect the seasonal occurrence of dolphins described by fishermen and according to the fishing season (Fig. 2). In experimental set nets, Aquatec Aquamark® 200 (www.aquatecgroup.com) pingers (wideband frequency modulated waveforms, 200-300 m long, with harmonic energy in the 5 kHz to 160 kHz band, typically 145 dB re 1 μPa at 1 m) were also tested. To quantify net damage, identical orange multifilament trammel net sections were used, each measuring 1.2 m in height and 80 m in length (total net area: 96 m^2^). These trammel nets had an internal mesh size of 18 mm^2^ and an external mesh size of 100 mm^2^ (Fig. 3). As they are relatively indiscriminate, these nets can be employed for a wide variety of target catch, most typically red mullet species *Mullus spp*. The nets cost €155 (220 USD) each in 2010.

During each experimental set, two trammel net sections were set at opposite ends of a continuous line of other set gillnet and/or trammel net sets, deployed by participating fishers, measuring hundreds to thousands of meters (Fig. 3, see insert). One of these two 80 m long trammel net sections had a pinger attached to the float line at each end (Fig. 3), the other did not. By setting nets in this semi-independent/paired way with commercial sets, typical commercial fishing conditions were replicated while the impact of any random effects such as depth (nets are set parallel to bathymetric contours), target catch, vessel captain, other depredating animals such as sea turtles, invertebrates etc., were minimised. As the commercial sets involved in our experiments totalled many hundreds of meters in length, the control net was set at sufficiently distant from the pinger net, and greater than the manufacturer’s advised spacing of 200 m for these devices. The distance between pinger and control nets was 725 m ± 56 m (mean ± SE). Target catch, depth, and placement of the set were at the discretion of the participating fisher, but the experiment was not undertaken when fishing targeted siganids (Rabbitfishes) in shallow water since dolphin depredation was considered by fishers to be less important there. Target species were striped red mullet (*Mullus surmuletus*; *n* = 25 sets; 54.3%), bogue (*Boops boops*; *n* = 15 sets; 32.6%), Mediterranean parrotfish (*Sparisoma cretense*; *n* = 3 sets, 6.5%), and picarel (*Spicara smaris*; n = 3 sets, 6.5%). To limit cumulative damage to the experimental trammel net sections, three identical labelled pairs of nets were used in rotation. Prior to deployment, a coin toss was used to decide which of the two nets of each pair would serve as the experimental unit. This was to reduce biases that might confound results from, for example, a net repeatedly subjected to the same treatment incurring more cumulative damage and subsequently catching fewer fish and resulting in fewer dolphin interactions. A coin toss was also used to allocate which of the two experimental nets would be deployed first, to avoid biasing treatments due to any resulting modest difference in soak time. Set depths were recorded to the nearest meter at the beginning of each control and pinger net set from an on-board acoustic fish sounder (Table 1). To estimate the presence of dolphins around sets (Leeney *et al.* 2007), each control and pinger net was set with one CPOD moored at the terminal end at the bottom of the anchor line and stationed approximately 2 m above the sea bed (Fig. 3). CPOD datasets were gathered from 44 control nets and 45 pinger nets.

Damages to the internal mesh panel of each of the two experiment nets were recorded after every experiment by counting the number of internal mesh units that were removed or damaged. The area of each mesh unit was 324 mm^2^ (18 * 18 mm), so we quantified the extent of damage as the number of affected mesh units multiplied by the mesh unit area. Once recorded, damage was labelled using adhesive tape, thus allowing the differentiation of damage occurring during successive experiments. On hauling, all fish were removed, identified, and weighed individually, except for on one occasion when a large haul of *Spicara smaris* was weighed in total to the nearest kilogram to prevent it spoiling, since saleable catch was returned to the fisher.

During trips, fishers often set other nets in addition to those involved in our experiments. The combined length of all net sets made during a sample of 27 field trips were measured (excluding the two 80 m experimental sets) using a handheld GPS.

Wilcoxon matched pairs signed-rank test was used (Wang *et al*. 2010) to compare dolphin presence (dolphin detection positive minutes), net damage (m^2^) and catch (g) between pinger and control nets and to compare damage between dolphin present and dolphin absent sets.

## Results

### Occurrence of Dolphins in Fishing Grounds

Ninety-two percent of fishers (*n* = 131 respondents) reported having observed dolphins in their fishing grounds at some point during the past year. More than 40% of fishers were active throughout the year and dolphins were observed during all months (*n* = 101 respondents; Fig. 2). The proportion of respondents that were active was greatest during spring-autumn peaking in May and the reported temporal pattern of dolphin encounters largely corresponded to their activity (Fig. 2).

**Fig. 4:**
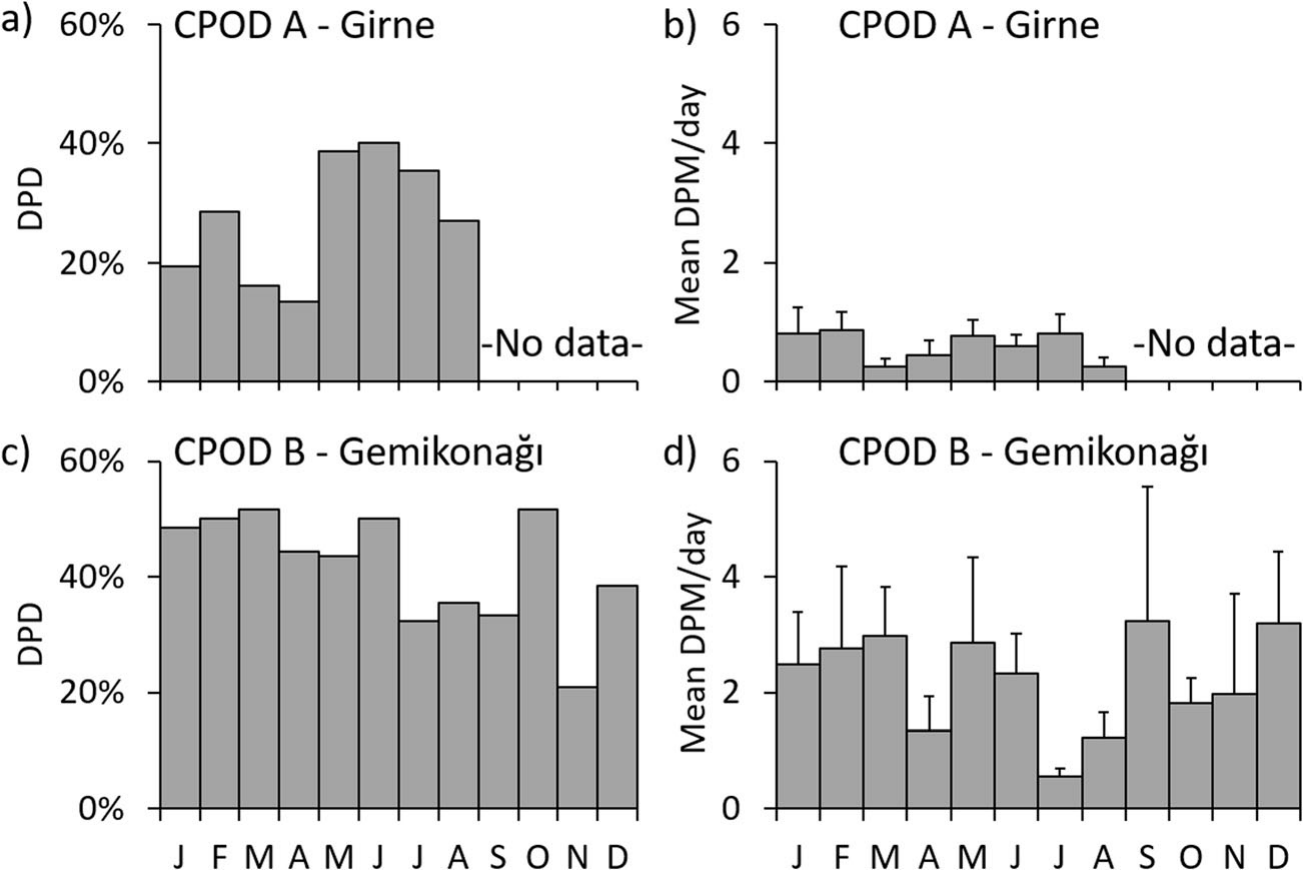
Summarised dolphin detections (Quality: Hi and Mod) at acoustic monitoring points by month during 2014. Proportion of dolphin detection positive days (DPD) in each month at a) CPOD A and c) CPOD B. Mean (with SE bars) proportion of detection positive minutes (DPM) per day by month at b) CPOD A and d) CPOD B. See Fig. 1 for locations of CPODs A and B. CPODs were not set at site A during September to December

**Fig. 5:**
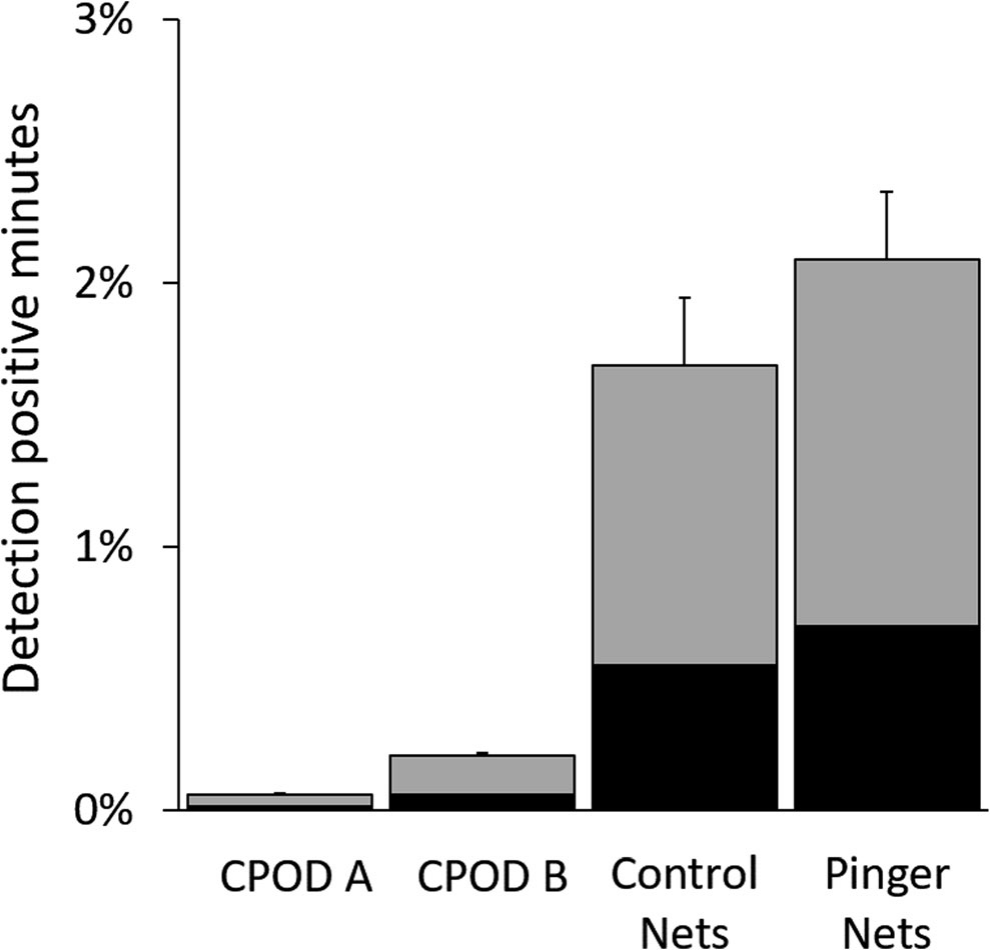
Proportion of dolphin detection positive minutes (DPM ± 95% CI) recorded at CPODs A, B, and CPODs at control and pinger net sets. Black and grey shading indicate high and moderate (respectively) quality assigned by KERNO classifier

Dolphin click trains were detected during all deployment months at CPOD monitoring points A and B (Fig. 4), confirming the year-round occurrence described by fishers. The proportion of dolphin detection positive days per month ranged from 13% to 52% with 0.3 to 3.2 dolphin detection positive minutes (DPM) per day (mean per month). During each month, frequency of dolphin detection positive days (Fig. 4a, c) and mean dolphin detection positive minutes per day (Fig. 4b, d), were higher at CPOD B (except July). Overall, DPM was higher at site B (0.21%) than at site A (0.06%; Fig. 5).

### Rate of Dolphin Interactions

Eighty-six percent (*n* = 87 respondents) of fishers used set nets. Fifty percent of fishers (*n* = 128 respondents) said that on the occasions they had observed dolphins, the dolphins had always damaged their nets, 41% said that the dolphins damaged their nets on some of the occasions on which they were observed, and 9% said that the dolphins had not damaged their nets on the occasions on which they were observed.

Bottlenose dolphins were visually confirmed during two experimental sets. Dolphins were detected by CPODs during 12 of the 45 experimental sets in which they were deployed, and were visually confirmed on one occasion where CPODs were not used. Dolphins were thus recorded during 13 (28%) of 46 experimental sets. The proportion of DPM was more than tenfold higher at CPODs associated with nets (control and pinger: 1.9%) than at CPODs A and B (0.2%; Fig. 5).

### Net Damage

**Fig. 6:**
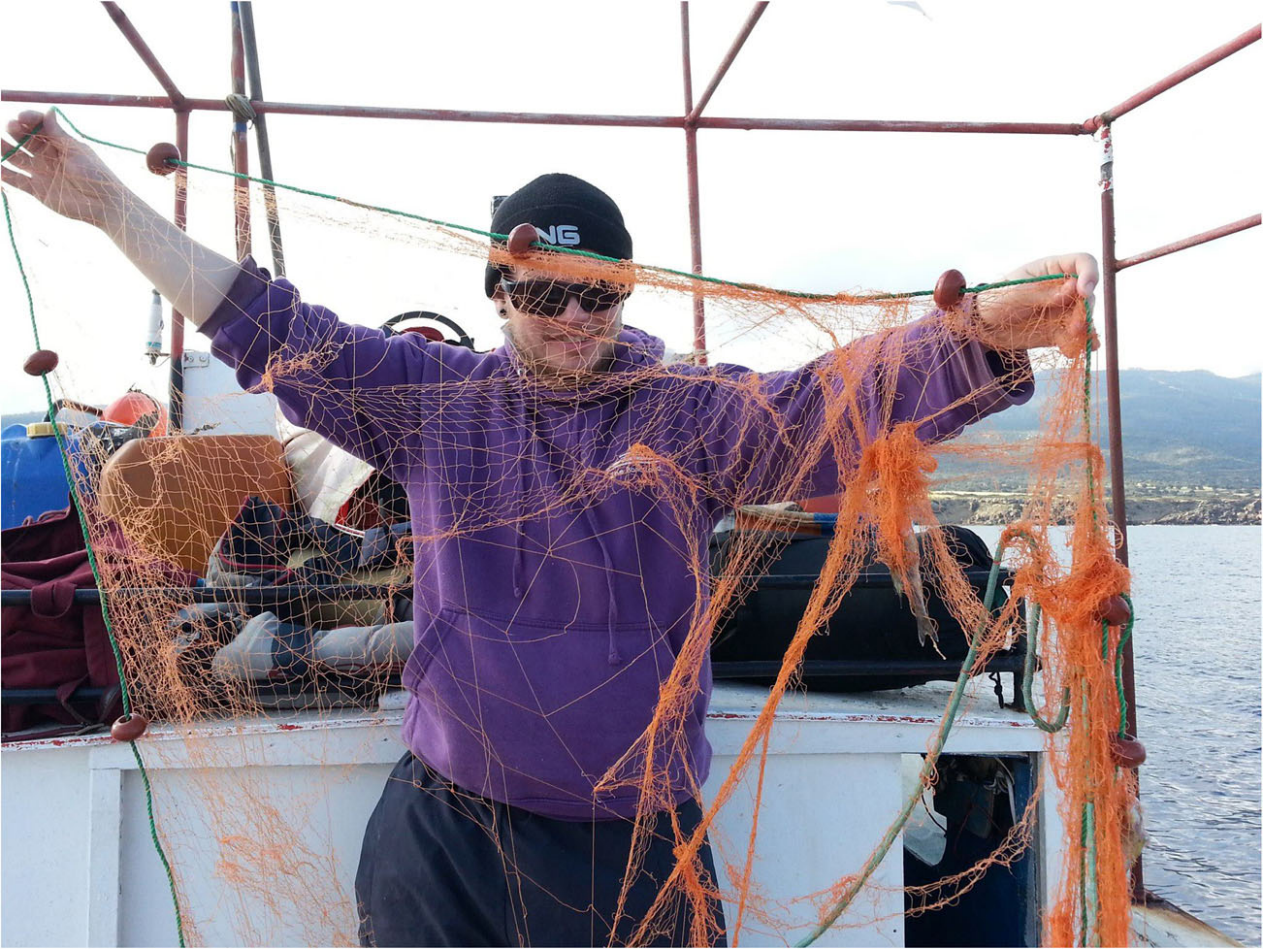
Experimental net damaged during a set at which dolphins were detected by CPODs

**Fig. 7:**
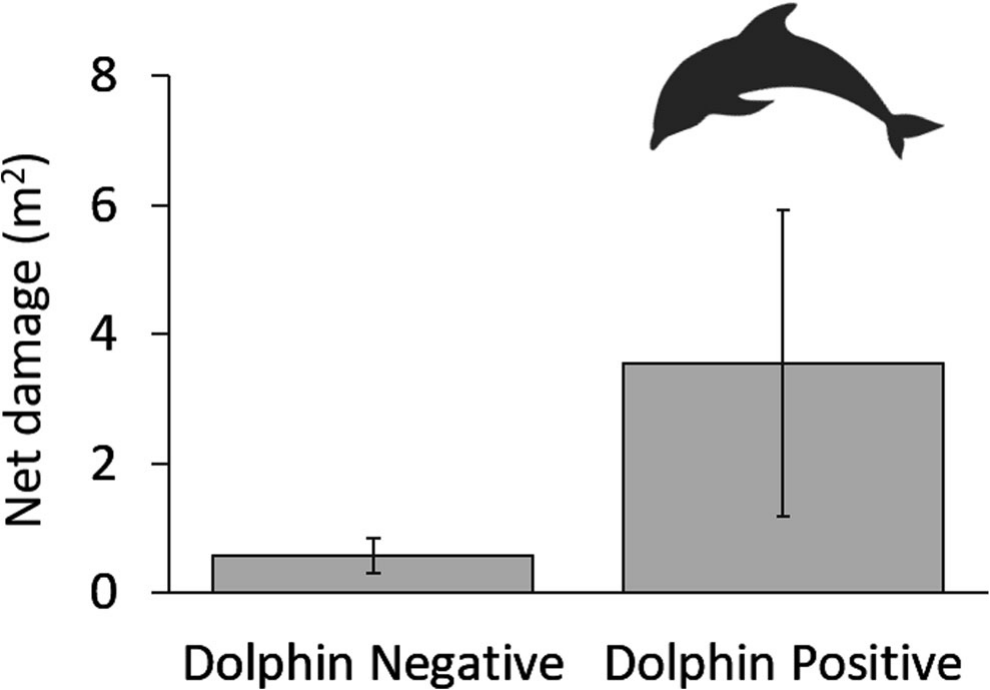
Damage to set nets (mean ± SE bars) for dolphin negative (no dolphins recorded at sets; 0.57m^2^ ± 0.27) and dolphin positive (dolphins recorded at sets; 3.55m^2^ ± 2.37) sets. Wilcoxon signed-rank test result: *P* = 0.08

Mean net damage per set for all sets of all treatments was 1.4m^2^ per 80 m set (SE: ± 0.7, range: 0.0–63.1) or a loss of 1.5% of the total net area. However, damage to nets was six times greater where dolphins were recorded (mean ± SE: 3.6m^2^ ± 2.4; 3.7% area vs. 0.6m^2^ ± 0.3; 0.6% area; Fig. 6). Although these differences were found to be non-significant (*P* = 0.08; Fig. 7), net damage was significantly correlated with the proportion DPMs recorded at CPODs during sets (Spearman’s correlation coefficient test statistic: rho = 0.26, *P* = 0.02; with outliers removed rho = 0.22, *P* = 0.04) suggesting that dolphins were a driver of net damage.

### Pinger Effect

There was no significant difference in the amount of net damage (*P* = 0.26), DPM (*P* = 0.56) or haul mass (*P* = 0.85) among pinger and control sets (Table 1).

### Fishing Effort and Landings

Of 135 respondents, 19% made 1–11 trips, 50% made 11–20 trips, 10% made 21–30 trips and the rest made over 30 trips per month. The experimental nets caught 98.1 kg of fish during the 46 experiments. Mean haul mass was 1.1 kg per 80 m set (SE: ± 0.18; range: 0–10 kg). Fifty-seven species were caught, the majority in small amounts and not saleable locally. Of 21 species where the total catch from all sets exceeded 500 g (Fig. S1), 10 species made up the saleable catch, which were 78% of the total catch, the remaining 22% were used for bait, subsistence, or were discarded.

The mean combined length of nets set by fishers per observed trip (*n* = 27) was 2004 m (SE: ± 148, range: 781–4150). By extrapolation using the mean observed haul per 80 m set and the mean combined set length we estimated that on average 26.7 kg of fish are caught in set nets per fishing trip. For fishers making 132–240 trips per year (most fishers make 11–20 trips per month; see above) annual catch in set nets is therefore 3.5–6.4 t/year. Assuming 215–300 active vessels (estimated by Snape *et al*. 2013), the total catch of the northern Cyprus set net fishery is therefore between 759 and 1923 t/year (215 vessels landing 3.5 t/year to 300 vessels landing 6.4 t/year).

### Group Sizes and Bycatch

Group sizes of two to three were estimated on each of the two occasions when onboard observers sighted dolphins and, on both occasions, they were identified as bottlenose dolphin (*Tursiops truncatus*). These estimates support those of fishers who reported seeing groups of 1–5 dolphins (62%), with others seeing groups of 6–10 (24%) and > 10 (14%; *n* = 114 respondents). Two fishers (1.7%; *n* = 118 respondents) had caught one dolphin during the preceding year, both dolphins were dead on hauling. Extrapolating this result to the number of fishing vessels that were active during the study (215–300 estimated for the study area by Snape *et al*. 2013), an indicative annual dolphin mortality level is likely to be less than 10 per year. No dolphin bycatch was recorded during onboard observation.

## Discussion

The use of CPODs to record dolphin occurrence over time during monitored set net sets is novel and has potential for standardising cetacean-fisheries interaction studies to allow more meaningful comparisons among fisheries, regions, and dolphin populations. Such techniques are increasingly being used for the detection of a range of cetacean species (Baumann-Pickering *et al*. 2015; Hardy *et al*. 2012; Miller *et al*. 2015). Estimates of depredation rates vary across the Mediterranean. During the current study, dolphins were recorded at 28% of sets, greater than a 12.4% rate of depredation reported in Corsica (Rocklin *et al*. 2009), less than a 38% rate of depredation off Sicily (Buscaino *et al*. 2009) and the 68.7% rate reported in Sardinia (Díaz López 2006). Although the relatively low number of experimental sets limited the statistical power of our analysis, the occurrence of dolphins was far greater at set nets than at control sites, there was greater damage to sets at which dolphins were recorded and a positive correlation between acoustic detections and net damage. The latter is a useful result in that for further studies, CPOD data may possibly be used as a yardstick to measure economic losses during sets, without the necessity to laboriously mark and count damage incurred.

### What Is the Cost?

Net damage resulting from dolphin depredation can certainly be very costly to fishers. During one set when dolphins were present (42% DPM for the set duration and visually confirmed during hauling), an experimental net lost 79% of its area and was beyond repair. To quantify the true costs of net damage, a more detailed study of the economics of set-net fishing is required because the mechanisms used by fishers to address net damage are not known. However, our study provides evidence to support claims of Mediterranean fishers that they are expending thousands of Euros annually replacing nets. The mean set net length of 2004 m costs >€3000; during the current study 80 m sections cost €155 and were competitively sourced. At the observed mean damage rate of 1.5% area per set, most fishers, who we estimate to be making 132–240 sets per year, may need to address their net damages many times annually if, hypothetically, they replace their nets once 50% area damage is reached. Compounding these costs, fishers increase their set lengths over time, to maintain catch rates against decreasing fish stocks (Ulman *et al*. 2014). Additionally, loss of earnings due to spoiled catch through depredation, although of secondary importance to the fishers we interviewed, likely also constitutes significant loss of earnings (Lauriano *et al*. 2009). Fish landing prices across Mediterranean countries of the European Union are relatively high (EC 2002) and in Cyprus are the highest (data for southern Cyprus; GFCM 2016) and dolphin depredation may be of influence here, such are the cumulative costs of landing fish.

### Dolphin Population Insights

The passive acoustic monitoring results presented here for reference sites show that dolphins are present off the coast of Cyprus throughout the year. In Atlantic and Pacific waters, passive acoustic monitoring also found year-round presence of bottlenose dolphins (Simon *et al*. 2010; Elliott *et al*. 2011). These studies, however, used arrays of TPODs (precursor of the CPOD) and detected seasonal and spatial patterns in dolphin behaviour. Longer-term studies using CPOD arrays would be useful in assessing the habitat use of dolphins using the coast of Cyprus (Kiszka *et al*. 2012) to examine in more detail their seasonal interaction with fisheries (Blasi *et al*. 2015) and to estimate more accurately the true threat level from fisheries and other sources (Hashimoto *et al*. 2015; Huang *et al*. 2014; Parsons *et al*. 2015).

Our indicative dolphin mortality estimate of <10 individuals per year, demonstrates that bycatch is an occasional occurrence in northern Cyprus. This could be considered conservative, since fishers may withhold information regarding mortality of protected species. Appreciable quantities of net were removed from our study nets by dolphins and laryngeal strangulation (Gomerčić *et al*. 2009) or gastro-intestinal complications associated with consuming net pieces, could be a secondary source of mortality. Given the small group sizes reported, such losses are a cause for concern (Brotons *et al*. 2008b). To understand the threat level in a population context, more extensive onboard observations are required to fully assess the bycatch rate, whilst boat-based cetacean surveys, aerial survey, telemetry studies, and strand monitoring would be useful in assessing dolphin density, seasonal movements, and population connectivity (Mullin *et al*. 2017; Byrd and Hohn 2017).

### Pervasive Fishing Increasingly Driving Dolphin Depredation

Whilst investigating the conflict between Mediterranean SSF and bottlenose dolphins, this study also provides a useful insight into the nature and intensity of a fishery for which very few data exist. Since northern Cyprus is a de facto state, no fisheries landing statistics are recorded by the FAO. This is the subject of a recent study (Ulman *et al*. 2014) that estimated the most active 11% of fishers were landing just 2.7 t/year in 2013 and that the majority landed <0.2 t/year. The results of the current study suggest that the majority of fishers may land 3.53 t annually in set nets alone. Mean annual landings for Cyprus of 1749 t (2010–2013) reported by the General Fisheries Commission for the Mediterranean (GFCM 2016) are likely to be underestimated by at least 40%, given the minimum catch estimate for northern Cyprus set net fisheries estimated here. It is therefore important to incorporate first-hand on-board observations into catch estimates. Although little catch goes to waste, the sustainability of this fish extraction must be considered as the ecosystem impacts of continuous removal of fish are acute and are considered a driver of dolphin depredation (Read 2008; Rocklin *et al*. 2009).

Over-fishing in the Mediterranean has become so pervasive that it has created a vicious cycle for the dolphins and fishers as they compete for the remaining fish. The depleted fish stocks result in extremely low catches, requiring more net and a situation where depredation can result in significant economic losses because any loss is significant. Depleted prey for the dolphins appears to fuel the depredation problem and perhaps explains why the acoustic pingers, which are designed to repel dolphins, have instead attracted them in some cases. Given the dolphin depredation reported here, the intensity of sound emitted by our acoustic sounding pinger (max 145 dB) may have been insufficient to act as deterrent.

### Future Management Implications

Higher intensity acoustic pingers, termed mid-range or acoustic harassment devices by Dawson *et al*. (2013), could be tested and user-programmable deterrents are available. However, if high intensities are found to be more successful, the impact of introducing high intensity anthropogenic noise into coastal fishing grounds must also be properly assessed to avoid unintended consequences (Parsons *et al*. 2015). Aside from the impact of excluding dolphins from foraging areas, there may be adverse impacts on other species (Estabrook *et al*. 2016; Hatch *et al*. 2016; McKenna *et al*. 2016), particularly cetaceans, although published data regarding this group are scarce in the region (Reeves and Notarbartolo Di Sciara 2006).

A more holistic managerial approach should be urgently developed not only to provide dividends for northern Cyprus’s coastal ecosystems, but for fishers too, as sustainably managed stocks support more profitable fisheries (Hilborn 2012; Quetglas *et al*. 2016; Vendeville *et al*. 2016). Currently, fishing using set nets in northern Cyprus is unlimited to licenced fishers, with no restricted zones, periods, size selectivity or landing quotas, and no assessment or monitoring of stocks. Fishers from across northern Cyprus have called for sustainable management and cite lack of government capacity to develop and regulate restrictions. Such governance problems are common among small-scale fisheries, where bottom-up management of stocks are increasingly being employed, with benefits including increased and stabilised landings and landing prices and improved catch per unit effort (Beger *et al*. 2004; Defeo *et al*. 2014; Gelcich *et al*. 2009). Northern Cyprus could serve as an arena to test management models in which sustainable systems are managed by fishers (Tilman *et al*. 2017) and once established, these systems may result in reduced dolphin interactions, since overexploitation drives depredation. Given that the revised Common Fisheries Policy aims to ensure that all commercial fish stocks are managed at their maximum sustainable yield by 2020 (Chato Osioa *et al*. 2015), the European Union could be called upon with priority to build capacity in fisheries management and governance, for example through its aid programme for the Turkish Cypriot Community (EU regulation No. 389/2006: http://eur-lex.europa.eu/legal-content/EN/TXT/PDF/?uri=CELEX:32006R0389&from=EN).

## Electronic supplementary material

Supplementary figure S1 Total haul mass (kg) across the 92 experimental sets of 80 m trammel nets. Only species for which total haul mass exceeded 0.5 kg are shown. (PNG 24 kb)

## Footnotes

1 For example sighting of dolphins at sets: Brotons *et al*. 2008a; Gazo *et al*. 2008; in fishing areas: Bearzi *et al*. 2011; Blasi *et al*. 2015; net damage, various methods: Brotons *et al*. 2008b; Gazo *et al*. 2008; Rocklin *et al*. 2009; damaged catch: Brotons *et al*. 2008a; Rocklin *et al*. 2009; damaged catch on sighting dolphins: Gazo *et al*. 2008; total landings: Brotons *et al*. 2008a; Buscaino *et al*. 2009; catch composition: Rocklin *et al*. 2009.
