# Supplementary figures and images for: Conflict between Dolphins and a Data-Scarce Fishery of the European Union
Source: Hum Ecol Interdiscip J. 2018 Mar 27;46(3):423–33. doi: 10.1007/s10745-018-9989-7 (PMC6015611; doi:10.1007/s10745-018-9989-7)

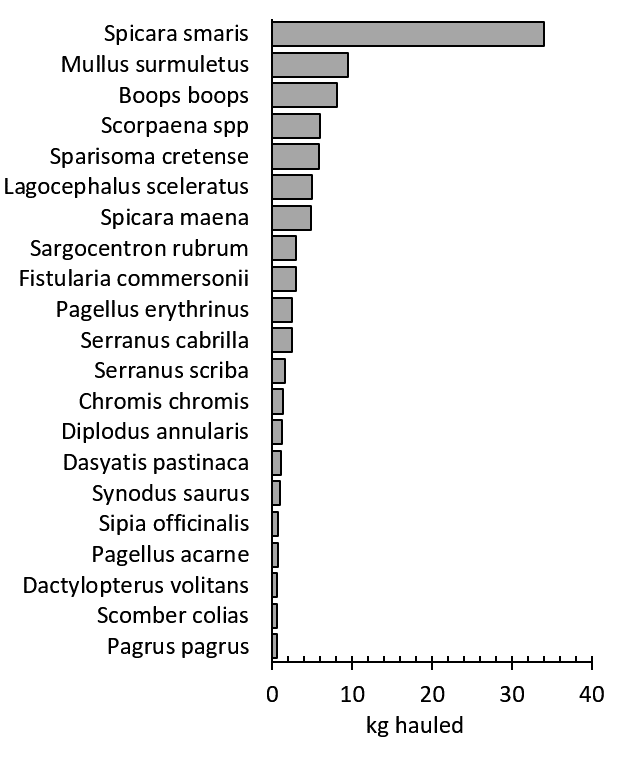

Supplement: Supplementary file 1 — Total haul mass (kg) across the 92 experimental sets of 80 m trammel nets. Only species for which total haul mass exceeded 0.5 kg are shown. (PNG 24 kb) [file 10745_2018_9989_MOESM1_ESM.png]
